# Advancing Construction 3D Printing with Predictive Interlayer Bonding Strength: A Stacking Model Paradigm

**DOI:** 10.3390/ma17051033

**Published:** 2024-02-23

**Authors:** Dinglue Wu, Qiling Luo, Wujian Long, Shunxian Zhang, Songyuan Geng

**Affiliations:** 1 College of Civil and Transportation Engineering, Shenzhen University, Shenzhen 518060, China; luoqiling@szu.edu.cn (Q.L.); longwj@szu.edu.cn (W.L.); 2 Guangdong Provincial Key Laboratory of Durability for Marine Civil Engineering, Shenzhen Key Laboratory for Low-Carbon Construction Material and Technology, Shenzhen 518060, China; 3 Key Lab of Coastal Urban Resilient Infrastructure, MOE, Shenzhen 518060, China; 4 Poly Changda Engineering Co., Ltd., Guangzhou 510620, China; 13926060209@163.com (D.W.); shunxian22@126.com (S.Z.)

**Keywords:** intelligent prediction, 3D printing concrete, interlayer bonding strength, machine learning, stacking strategy

## Abstract

To enhance the quality stability of 3D printing concrete, this study introduces a novel machine learning (ML) model based on a stacking strategy for the first time. The model aims to predict the interlayer bonding strength (IBS) of 3D printing concrete. The base models incorporate SVR, KNN, and GPR, and subsequently, these models are stacked to create a robust stacking model. Results from 10-fold cross-validation and statistical performance evaluations reveal that, compared to the base models, the stacking model exhibits superior performance in predicting the IBS of 3D printing concrete, with the *R*^2^ value increasing from 0.91 to 0.96. This underscores the efficacy of the developed stacking model in significantly improving prediction accuracy, thereby facilitating the advancement of scaled-up production in 3D printing concrete.

## 1. Introduction

Three-dimensional printing technology constructs physical objects layer by layer from digital models, offering unparalleled design flexibility, rapid prototyping capabilities, and efficient production processes [1]. Capitalizing on its inherent advantages, 3D printing technology has found widespread applications across various fields [2]. When applied to the realm of architecture, it holds immense potential to streamline prototype design and enable the creation of complex structures with heightened efficiency [3]. However, 3D printing technology has not yet gained widespread adoption in the field of architecture, primarily due to the unstable quality of printed structures [4]. The characteristic layer-by-layer manufacturing process of 3D printing results in a lack of fusion between layers, creating critical weak points commonly referred to as “cold joints” [5]. These “cold joints” can lead to instability in the strength and stiffness of the structure, consequently reducing its load-bearing capacity [6]. Therefore, ensuring the sufficient strength and durability of the structure requires achieving robust interface bonding and maintaining the interlayer bonding strength (IBS) at a higher stable level. To precisely understand and promptly adjust the IBS of 3D printing concrete, researchers have explored various adjustment methods. For example, Wolfs et al. [7], employing physical mechanics experiments, identified the impact patterns of three printing parameters—printing layer interval time, printing nozzle height, and printing layer surface moisture content—on the IBS of 3D printing concrete. According to these patterns, adjustments were made to the IBS of 3D printing concrete. In addition to physical mechanics testing methods, Moelich et al. [8] utilized computer simulation techniques to propose an analytical model for characterizing the relationship between surface moisture and the IBS of 3D printing concrete. Based on this model, optimizations were made to the IBS of 3D printing concrete. However, both physical mechanics testing methods and computer simulation techniques have some inevitable drawbacks. The former may result in substantial time and resource consumption while struggling to find a globally optimal solution. The latter’s high dependence on parameter settings may reduce the accuracy and stability of simulation results.

Data-driven machine learning (ML) algorithms enable computers to learn patterns and make predictions or decisions, excelling in handling massive amounts of data, identifying complex patterns, and continuously improving performance over time [9]. Integrating ML algorithms into the field of architecture, such as predicting the basic properties of concrete, can enhance construction efficiency, reduce costs, and promote environmental conservation [10]. Some researchers have already employed ML algorithms for modeling concrete performance prediction. For instance, Koya et al. [11] compared the predictive performance of five ML algorithms for the mechanical properties of ordinary concrete. Bui et al. [12] and Shahmansouri et al. [13], respectively, utilized ML algorithms to predict the compressive strength of high-performance concrete and geopolymer concrete, demonstrating their effectiveness. Despite the numerous advantages of base ML algorithms, the limited availability of actual research data on concrete performance may constrain the accuracy and generalization ability of model training [14]. Stacking, as an ML ensemble strategy, has the potential to address these challenges. By combining multiple models, a stacking model often achieves better performance than a single base model. This is because it can leverage the strengths of each base model, thereby delivering superior performance in different aspects [15]. Li and Song [16] investigated the effectiveness of the stacking model in predicting concrete compressive strength and compared the performance differences between the base and stacking models. The research results indicate that the performance of the stacking model significantly surpasses that of the base model.

The early-stage methods for controlling the IBS of 3D printing concrete involve significant investments in terms of resources, manpower, and time, with the potential challenge of not achieving a global optimum solution. With the increasing prominence of the unique advantages of ML algorithms, there is an opportunity to incorporate them into the prediction of the IBS of 3D printing concrete. This entails evaluating the printing outcomes in advance, thereby assisting in avoiding unnecessary physical experiments. However, current research on predicting the performance of 3D printing concrete using ML algorithms is relatively limited, with only a few scholars attempting to predict parameters such as compressive strength [17], flexural strength [18], tensile strength [18], rheological properties [19], printability [20], etc., while no one has yet used ML methods to predict IBS. IBS is a critical parameter determining the quality of 3D printing concrete, highlighting the necessity of its advance prediction. In predicting the IBS of 3D printing concrete, some scholars have established correlation models between printing parameters and IBS based on traditional experimental methods [7], while others have proposed prediction models for IBS based on numerical simulation methods [8], and still others have enhanced IBS through induced thermo-fluid dynamics techniques [6]. However, these research methods often fail to guarantee achieving a globally optimal solution, thus necessitating the intelligent prediction of IBS using ML algorithms. Within ML algorithms, stacking models can fully leverage the advantages of base ML models while mitigating their disadvantages, thereby enhancing overall prediction accuracy. Some scholars have already used stacking models to predict concrete performance, achieving promising results [16]. However, no scholar has yet applied stacking models, which have enormous application potential, to predict 3D printing concrete IBS. Therefore, the innovation of this study lies in establishing a stacking model aimed at objectively and effectively predicting the IBS of 3D printing concrete. This not only promotes the widespread adoption of 3D printing technology in construction but also holds the promise of contributing to sustainability and efficiency improvements within the architecture sector.

Before determining the base ML algorithms, various algorithms are attempted, including decision tree (DT), support vector regression (SVR), ridge regression (Ridge), k-nearest neighbor (KNN), Gaussian process regression (GPR), least absolute shrinkage and selection operator (Lasso), ordinary least squares (OLS), and extreme gradient boosting (XGBoost). In the evaluation process, to ensure the consistency of computational conditions, 10-fold cross-validation is employed to reduce the risk of model overfitting. After extensive computations, it is found that SVR, KNN, and GPR perform well in predicting the IBS of 3D printing concrete, while the remaining ML algorithms exhibit poor performance (*R*^2^ less than 0.5). Even when using optimization algorithms to tune their hyperparameters, the performance of these models may not reach satisfactory levels. Therefore, SVR, KNN, and GPR are ultimately selected as the base models for the stacking model. Consequently, this study is primarily based on the SVR, KNN, and GPR algorithms, and their ensemble stacking model, constructing a comprehensive and efficient 3D printing concrete IBS prediction model. Initially, relevant data are gathered from various reliable sources, resulting in the establishment of a database comprising 146 datasets. Subsequently, leveraging this database, training is conducted for three base models (SVR, KNN, and GPR) and one stacking model. In this process, a 10-fold cross-validation method is employed to enhance the predictive accuracy of the models, and a comparative analysis of model performance is conducted using statistical metrics. Finally, an evaluation of the contributions of various influencing factors to the prediction of the IBS of 3D printing concrete is conducted based on importance ranking. This comprehensive research framework provides substantial support for in-depth exploration in the field of 3D printing concrete. The main contents of this study’s sections are as follows. Section 1 serves as the introduction, outlining the advantages of 3D printing concrete technology, its key influencing factors, the application of ML methods in the concrete domain, advanced stacking strategies, research innovations, and major themes. Section 2 presents the methodology, explaining the principles behind stacking models, base models, the meta-model, k-fold cross-validation, statistical checks, and hyperparameter tuning. Section 3 delves into the ML modeling process, covering dataset information, the predictive performance of base and stacking models, and their comparative results. Section 4 focuses on the importance and SHAP analysis, elucidating the principles of key features significantly impacting 3D printing concrete IBS and their positive/negative contributions. Section 5, the discussion and future prospects, summarizes the study’s findings and offers prospects for future research directions. Section 6, the conclusions, summarizes the main discoveries and conclusions of this study.

## 2. Methodology

### 2.1. Stacking Strategy

Stacking, within the domain of ML, is an ensemble learning technique where multiple models, often diverse in type or trained on distinct subsets of the data, are amalgamated to enhance overall predictive performance [21]. The fundamental concept of stacking involves utilizing the predictions generated by individual base models as input features for a higher level model known as the meta-model. By doing so, stacking addresses the limitations of individual models by capitalizing on their complementary strengths. This powerful technique proves particularly beneficial for improving predictive accuracy, especially in scenarios where a single model may not perform optimally across the entire dataset.

### 2.2. Base Models

#### 2.2.1. SVR

SVR is an ML algorithm used for regression tasks. It belongs to the family of support vector machines (SVMs) and is particularly effective in handling non-linear relationships between variables [22]. SVR operates by mapping input features into a higher dimensional space through a kernel function. Subsequently, it seeks to identify a hyperplane that optimally captures the relationship between the input features and the target variable. This process is integral to SVR’s ability to model complex relationships and make accurate predictions in various types of datasets [23].

#### 2.2.2. KNN

KNN stands out as a straightforward yet powerful ML algorithm employed for tasks related to both classification and regression. For regression, it predicts the target value by averaging the values of its k-nearest neighbors [24]. The simplicity of the algorithm is evident in its absence of an explicit training phase. Rather than undergoing a dedicated training process, it retains the entire training dataset and calculates predictions during runtime by evaluating proximity in the feature space [25]. KNN is intuitive, easy to understand, and particularly useful when dealing with small to moderately sized datasets [26].

#### 2.2.3. GPR

GPR is a non-parametric ML algorithm used for regression tasks. It belongs to the family of Gaussian processes, which are probabilistic models that define distributions over functions [27]. In GPR, instead of predicting a single point estimate for each data point, the algorithm models the entire distribution of possible functions that could describe the underlying relationship in the data. GPR is particularly well suited for scenarios where uncertainty estimation is crucial [28].

### 2.3. Meta-Model—Linear Regression (LR)

In the context of stacking, a meta-model functions as a higher level model that amalgamates the predictions of multiple base models, thereby enhancing overall predictive performance. The key attributes of a good meta-model include the ability to effectively blend diverse predictions and generalize well to new data. The choice of the meta-model is crucial as it influences the ensemble’s capacity to capture complex patterns and enhance predictive accuracy. Commonly used meta-models include linear models like LR, decision tree (DT), random forest (RF), and neural networks [29]. The selection depends on the nature of the problem, the diversity of the base models, and the desired trade-off between interpretability and complexity. After a series of practical computations and referencing the relevant literature [30], this study adopts LR as the meta-model.

LR stands out as an advantageous choice for a meta-model in stacking. Its interpretability is noteworthy, offering clear insights into the relationships between input features and the target variable, a crucial factor for understanding the impact of various base models on the final prediction. The computational efficiency of LR becomes invaluable in scenarios where training time or computational resources are limited [31]. Furthermore, the model’s inherent ability to handle linear relationships aligns seamlessly with stacking’s linear combination of predictions. The characteristics of LR as a meta-model include its exceptional blend-ability, excelling in combining diverse base models’ outputs to effectively exploit their complementary strengths [32].

### 2.4. k-Fold Cross-Validation and Statistical Checks

k-fold cross-validation is a valuable technique in training stacking models, offering several advantages and ensuring robust model evaluation. The necessity of k-fold cross-validation arises from its ability to provide a more reliable estimate of a model’s performance. This is achieved by systematically partitioning the dataset into k subsets, training the model on k-1 folds, and validating the remaining fold [33]. In this study, the choice of k = 10 is motivated by several key considerations. Firstly, given the relatively small size of the dataset used in this research, a larger value for k is deemed suitable. Secondly, as the value of k increases, there is a proportional increase in the computational resources and time required. Conversely, a smaller k might lead to higher variance in training for smaller datasets. Striking a balance between variance and computational time and resources is essential. Many classic references [34,35,36] highlight the effectiveness of the 10-fold cross-validation method, positioning it as a classical choice. Additionally, through a series of practical computations, it is observed that k = 10 achieves a favorable equilibrium between variance and computational efficiency. Based on these considerations, this study adopts the 10-fold cross-validation method. The dataset is partitioned into 10 folds, with each iteration involving training on 9/10 of the data and validating on the remaining 1/10, allowing for a comprehensive assessment.

Moreover, the model’s effectiveness and accuracy can be evaluated through the application of statistical metrics. These metrics encompass the coefficient of determination (*R*^2^) and root mean square error (*RMSE*), as delineated in Equations (1) and (2).

(1) $$R^{2}=1-\frac{{\sum_{j=1}^{n}\left(p_{j}-t_{j}\right)}^{2}}{\sum_{j=1}^{n}\left(t_{j}-\bar{t_{j}}\right)}$$

(2) $$RMSE=\sqrt{\frac{\sum_{j=1}^{n}{\left(t_{j}-p_{j}\right)}^{2}}{n}}$$

In the equations, *t_j_* is the experimental value; *p_j_* is the predicted value; $\bar{t_{j}}$ is the target mean value; and *n* is the total number of instances used in modeling.

### 2.5. Hyperparameter Tuning

Grid search is a hyperparameter optimization technique that exhaustively searches the specified hyperparameter space to find the best combination of hyperparameters. Grid search attempts all possible parameter combinations and performs cross-validation for each combination to evaluate the model’s performance. It is typically combined with cross-validation to ensure that the selection of hyperparameters is not influenced by a specific data split.

**Figure 1:**
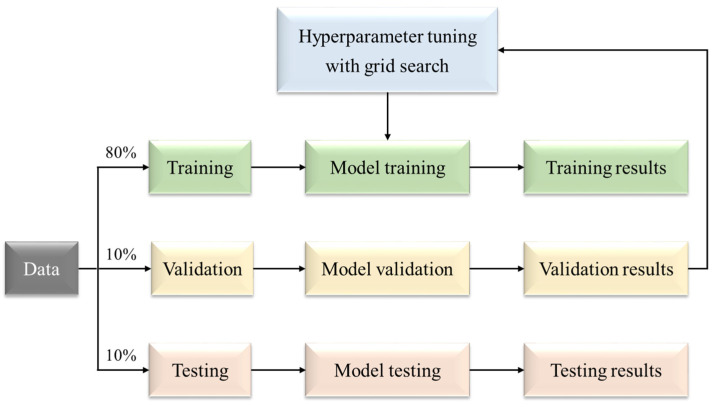
The evolution process of tuning hyperparameters.

Table 1 summarizes the definitions and ranges of hyperparameters for ML algorithms, where the ranges of hyperparameters are selected based on experimental and research experience from similar studies [37]. Figure 1 depicts the evolution process of tuning hyperparameters using 10-fold cross-validation and grid search. In this process, 80% of the data are used for training, 10% for testing, and 10% for validation [38]. In the process of hyperparameter tuning, a fine-grained grid search method is employed to gradually search for the optimal hyperparameters (the model has the lowest *RMSE*). The search intervals for hyperparameter values are determined based on previous research experience [39], as shown in Table 1.

### 2.6. Research Framework

**Figure 2:**
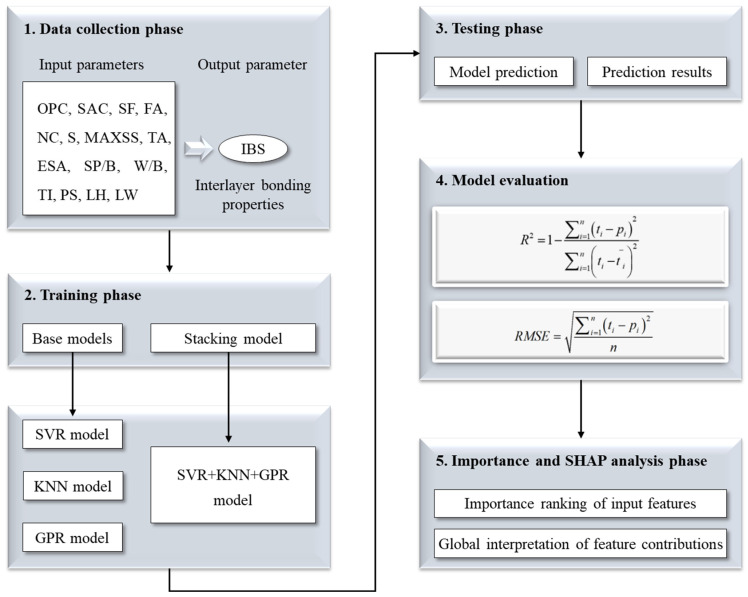
The general flow chart of this study.

The comprehensive workflow of this study is illustrated in Figure 2.

## 3. Modeling

### 3.1. Data Description

**Figure 3:**
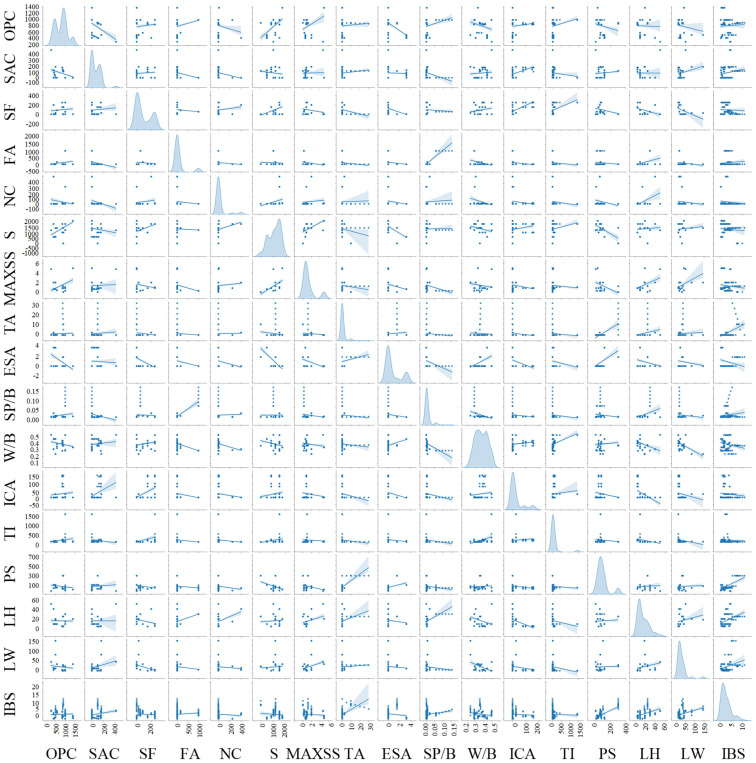
Pair diagrams showing variations of magnitudes of variables with each other from the model for predicting IBS.

**Figure 4:**
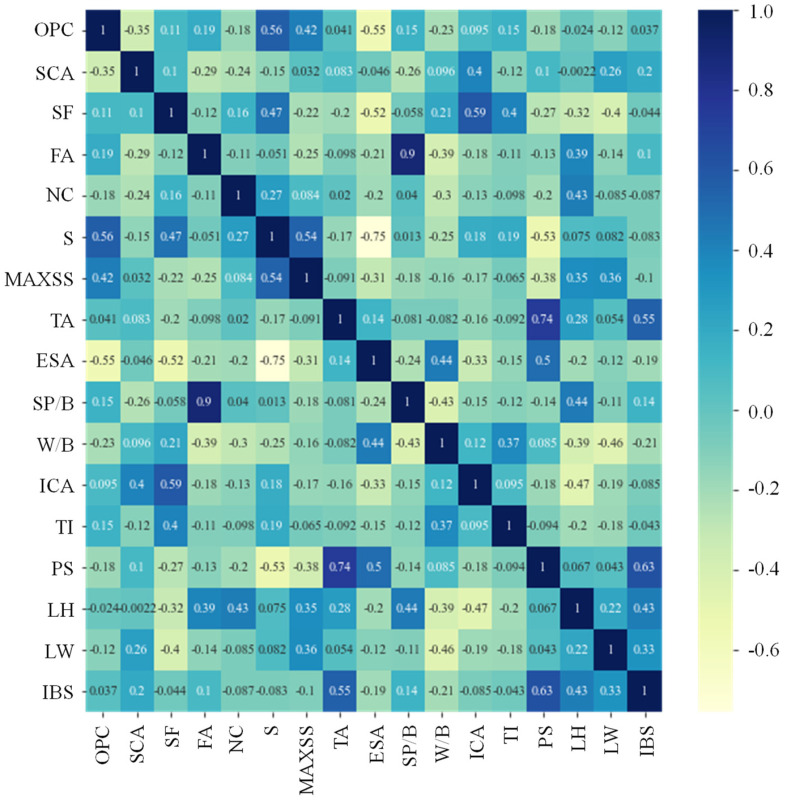
Correlation coefficients matrix from the model for predicting IBS.

To enhance the comprehensiveness and applicability of the prediction model, a total of 146 data points are collected from different studies [5,7,8,40,41,42,43,44,45,46,47,48,49] to establish a prediction model for the IBS of 3D printing concrete. The model incorporates sixteen input parameters, encompassing mix proportions (ordinary Portland cement—OPC; sulphate aluminate cement—SAC; silica fume—SF; fly ash—FA; nano clay—NC; sand—S; maximum sand particle size—MAXSS; thixotropic agent—TA; early strength agent—ESA; superplasticizer/binder—SP/B; water/binder—W/B; and interlayer bonding agent—ICA) and printing parameters (time interval—TI; printing speed—PS; printing layer height—LH; and printing layer width—LW). Table 2 lists the descriptive statistics of the data used to establish the prediction model (detailed data are shown in Table S1). In addition, Figure 3 shows the details of the pairing matrix as the variables change in value relative to each other. It is clear that the overall value of IBS decreases with increasing ESA and W/B, and increases with the increase of TA, PS, LH, and LW. The remaining variables do not have a significant effect on IBS. The results of the coefficient matrix are almost identical to the above results, as shown in Figure 4. For IBS, TA, PS, LH, and LW show strong positive correlations (0.55, 0.63, 0.43, and 0.33, respectively), and ESA and W/B show clear negative correlations (−0.19 and −0.21, respectively).

### 3.2. Hyperparameter Tuning and Independent Prediction Results of Base Models

**Figure 5:**
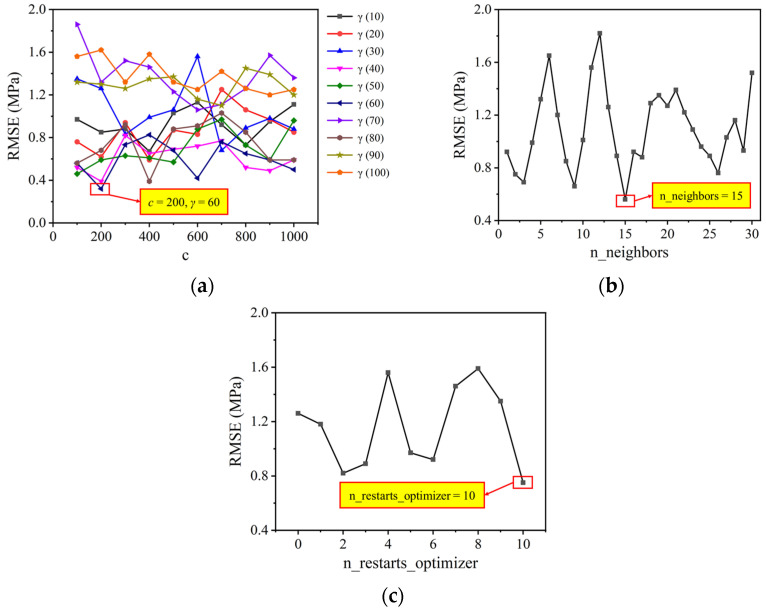
Evolution of *RMSE* for base models: (**a**) SVR; (**b**) KNN; and (**c**) GPR.

Following the methods outlined in Section 2.4 and Section 2.5, the evolution of *RMSE* through cross-validation for the SVR, KNN, and GPR models is obtained. As depicted in Figure 5a, the SVR model achieved the lowest *RMSE* when *c* = 200 and *γ* = 60. The optimal KNN model, as shown in Figure 5b, is obtained with n_neighbors = 15. According to Figure 5c, the GPR model performs optimally when the hyperparameter n_restarts_optimizer is set to 10.

**Figure 6:**
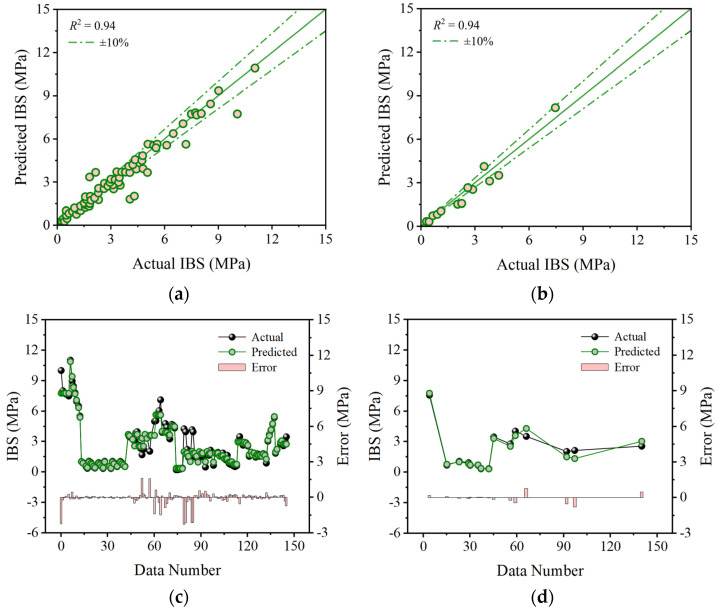
Comparison between predicted and actual IBS values from the SVR model: (**a**) correlation on the training set; (**b**) correlation on the testing set; (**c**) error on the training set; and (**d**) error on the testing set.

**Figure 7:**
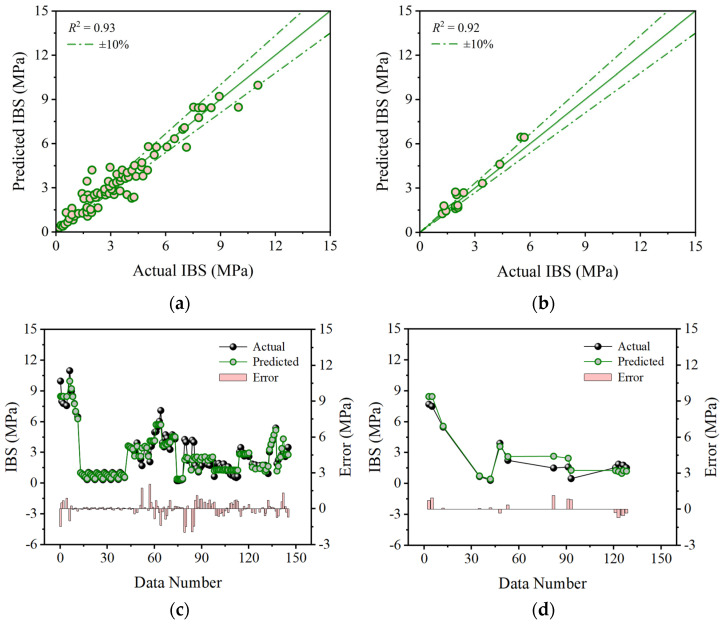
Comparison between predicted and actual IBS values from the KNN model: (**a**) correlation on the training set; (**b**) correlation on the testing set; (**c**) error on the training set; and (**d**) error on the testing set.

**Figure 8:**
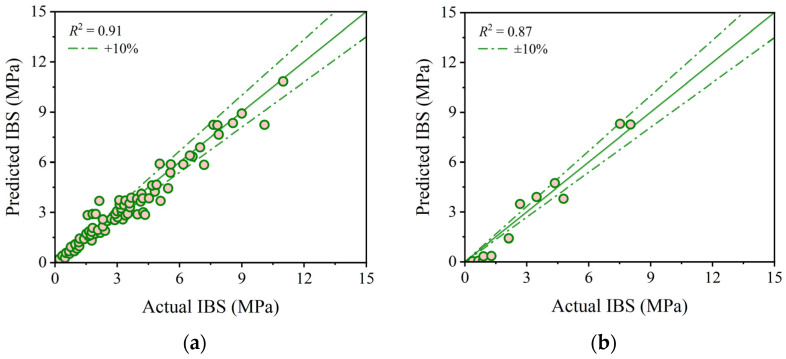

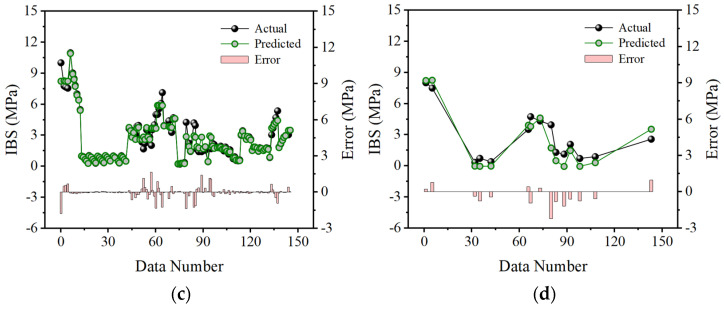
Comparison between predicted and actual IBS values from the GPR model: (**a**) correlation on the training set; (**b**) correlation on the testing set; (**c**) error on the training set; and (**d**) error on the testing set.

Based on the optimal hyperparameters, this study initially explores the training effects and testing results of base models in predicting the IBS of 3D printing concrete. Figure 6, Figure 7 and Figure 8 depict the prediction accuracy and errors of the SVR, KNN, and GPR models on both the training and testing sets. As shown in Figure 6, the SVR model achieves *R*^2^ values of 0.94 for both the training and testing sets, with mean errors of 0.27 MPa and 0.30 MPa, maximum errors of 2.27 MPa and 0.80 MPa, and minimum errors of 0 MPa and 0.02 MPa. Figure 7 reveals that the KNN model attains *R*^2^ values of 0.93 and 0.92 for the training and testing sets, accompanied by mean errors of 0.38 MPa and 0.52 MPa, maximum errors of 2.05 MPa and 1.14 MPa, and minimum errors of 0 MPa and 0.06 MPa. As illustrated in Figure 8, the GPR model shows *R*^2^ values of 0.91 and 0.87 for the training and testing sets, with mean errors of 0.67 MPa and 0.73 MPa, maximum errors of 1.78 MPa and 2.22 MPa, and minimum errors of 0 MPa and 0.22 MPa. The predictive results indicate that the aforementioned base models perform well, but there is still room for improvement. This necessitates the adoption of a stacking strategy in ML, leveraging the strengths of the base models to enhance overall predictive accuracy.

### 3.3. Hyperparameter Tuning and Prediction Results of the Stacking Model

Stacking the SVR, KNN, and GPR models, this study delves into their effectiveness in predicting the IBS of 3D printing concrete. Through the independent tuning and optimization of the stacking model’s hyperparameters, the optimal hyperparameters are determined: *c* = 500; *γ* = 20; n_neighbors = 16; and n_restarts_optimizer = 7.

**Figure 9:**
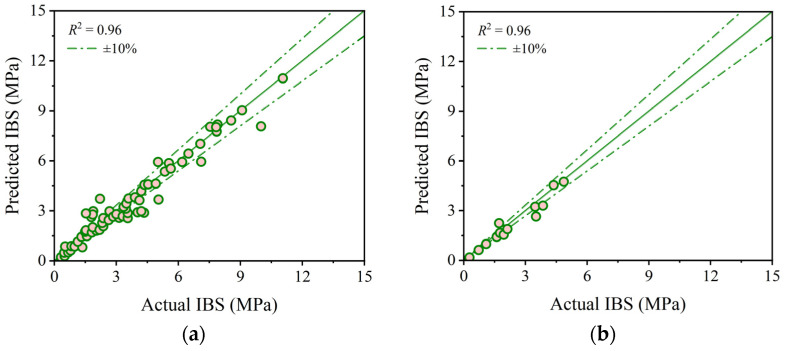

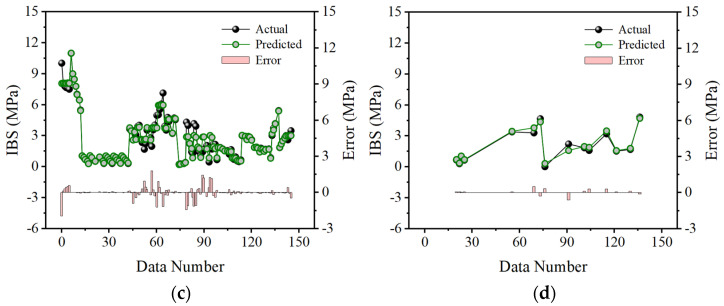
Comparison between predicted and actual IBS values from the stacking model: (**a**) correlation on the training set; (**b**) correlation on the testing set; (**c**) error on the training set; and (**d**) error on the testing set.

Figure 9 presents the predictive accuracy and errors of the stacking model using the optimal hyperparameters on both the training and testing sets. Visible in this figure, the stacking model achieves remarkable results on both sets, boasting *R*^2^ values of 0.96, mean errors of 0.22 MPa, maximum errors of 1.96 MPa and 0.63 MPa, and minimum errors of 0 MPa and 0.04 MPa. The performance of the stacking model on the training and testing sets remains similar, highlighting its robust generalization capability. Consequently, the stacking model demonstrates favorable overall predictive performance, showcasing outstanding capabilities and robustness in predicting the IBS of 3D printing concrete.

### 3.4. Comparison of Base and Stacking Models

**Figure 10:**
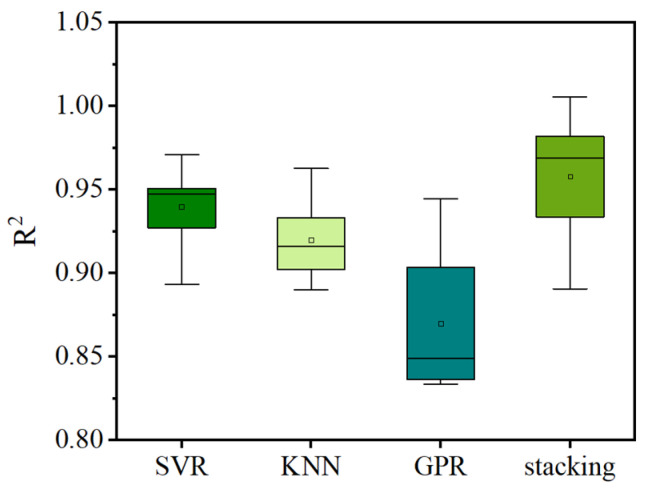
Box plot of prediction results for the base and stacking models obtained through 10-fold cross-validation.

*R*^2^ is selected as the primary evaluation metric for the results of the 10-fold cross-validation. Figure 10 displays a box plot illustrating the predictions of the base and stacking models for the IBS of 3D printing concrete through 10-fold cross-validation. From the figure, it is evident that the SVR model has an average *R*^2^ of 0.94, with a maximum of 0.97 and a minimum of 0.89. The KNN model exhibits an average *R*^2^ of 0.92, with a maximum of 0.96 and a minimum of 0.89. For the GPR model, the average *R*^2^ is 0.87, with a maximum of 0.94 and a minimum of 0.83. As for the stacking model, its average *R*^2^ is 0.96, with a maximum of 0.99 and a minimum of 0.89. Through comparative analysis, it is observed that, in the 10-fold cross-validation, the stacking model achieves the highest *R*^2^ and exhibits relatively minor fluctuation compared to the base models. This suggests that the stacking model attains the highest predictive accuracy and demonstrates a more stable performance compared to the individual base models.

The testing set contains data samples that the model has not encountered during the training process, allowing for an accurate evaluation of the model’s generalization ability to new samples. Consequently, this study places a key emphasis on a comparative analysis of the predictive metrics of the base and stacking models on the testing set, as outlined in Table 3. From the table, it is evident that, in comparison to the SVR, KNN, and GPR models, the stacking model demonstrates an increase in *R*^2^ by 2%, 4%, and 10%, respectively, while exhibiting a decrease in *RMSE* by 36%, 36%, and 55%, respectively. This improvement is attributed to the stacking of the three base models, enabling the amalgamation of their individual strengths. At this point, the negative effects of the base models are canceled out, ultimately improving the predictive performance of the stacking model.

**Figure 11:**
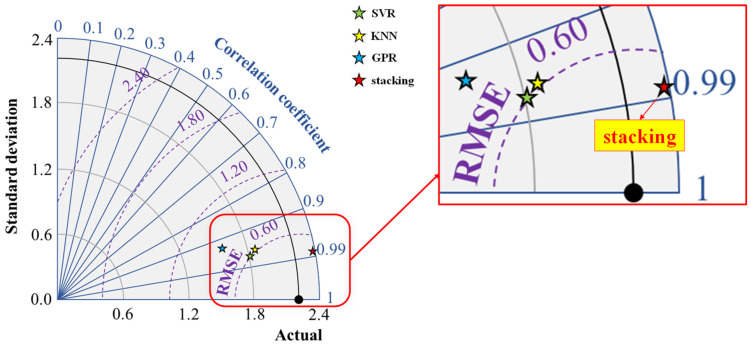
Taylor plot for comparing the performance of the base and stacking models.

Additionally, this study employs the Taylor plot to clearly illustrate the predictive performance and differences between the base and stacking models, as shown in Figure 11. The Taylor plot utilizes the correlation coefficient (*R*), *RMSE*, and standard deviation (*SD*) to quantify the differences between predicted and actual values, with the model being closer to the “Actual” point indicating better predictive performance. From the figure, it is evident that the stacking model is the one closest to the “Actual” point, reaffirming its superior performance in predicting the IBS of 3D printing concrete.

## 4. Importance Analysis and Shapley Additive Explanations (SHAP) Result

**Figure 12:**
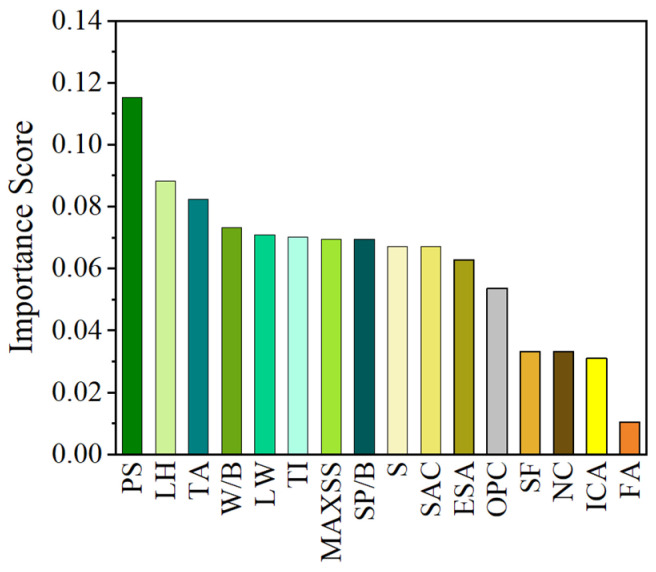
Importance ranking of factors affecting the IBS of 3D printing concrete.

In this study, a series of importance rankings for factors influencing the IBS of 3D printing concrete is obtained through the prediction of ML models. As shown in Figure 12, the top six factors, in descending order, are PS, LH, TA, W/B, LW, and TI. Among these factors, when compared to others, PS has the most significant impact on the IBS. A faster PS may lead to insufficient bonding between layers due to reduced deposition and curing time, resulting in a higher amount of uncured material and the potential weakening of interlayer bonding. Conversely, moderately reducing PS helps ensure a more thorough deposition and curing of each layer, increasing the robustness of interlayer bonding and forming a more uniform and reliable interlayer interface. Ranked second in terms of impact is LH. Adequate LH ensures the uniform deposition of concrete material for each layer and provides sufficient time for curing, thereby enhancing the robustness of interlayer bonding. However, excessive or insufficient LH may limit the full deposition and interaction of materials between layers, resulting in reduced IBS. Ranked third is the influence of TA, with the addition of an appropriate amount of TA contributing to improved concrete flowability. This facilitates easier deposition in each layer, enhancing the robustness of bonding between upper and lower layers. The introduction of TA also improves the viscosity and deformability of concrete, forming a more uniform and continuous structure at the interface between layers, which benefits enhancing bonding. However, an excessively high TA may lead to an excessive increase in concrete flowability, causing an unstable structure between layers and consequently reducing the IBS. The fourth-ranked factor in terms of impact is W/B. A moderate W/B ensures the sufficient flowability of concrete, promoting the formation of a tighter and more uniform interlayer interface, thereby increasing the IBS. However, an excessively high W/B may lead to an excessive increase in concrete flowability, causing an unstable structure between layers and reducing the IBS. A too low W/B may result in drier concrete, increasing the risk of cracking, which hinders material interaction between layers and consequently reduces the IBS. The impact of LW, ranked fifth, remains relatively significant. An appropriate LW contributes to the formation of a uniform and continuous interlayer interface, enhancing the IBS between printing layers. However, excessively large or small LW may lead to uneven material distribution and irregular interlayer interface shapes, reducing bonding surface area and consequently affecting the robustness of interlayer bonding. Finally, the sixth-ranked factor is TI. A suitable TI ensures the sufficient interaction of bonding surfaces, promoting the formation of a uniform and continuous interface and increasing the IBS. However, a too short TI may result in the incomplete curing of the previous layer, affecting the bonding of the next layer, while a too long TI may lead to fractures between adjacent layers, impacting the formation of bonding surfaces.

**Figure 13:**
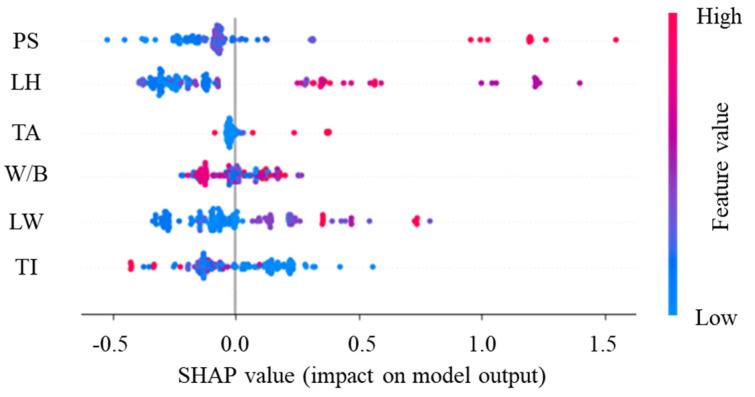
Global SHAP values for predicting the IBS of 3D printing concrete.

**Figure 14:**
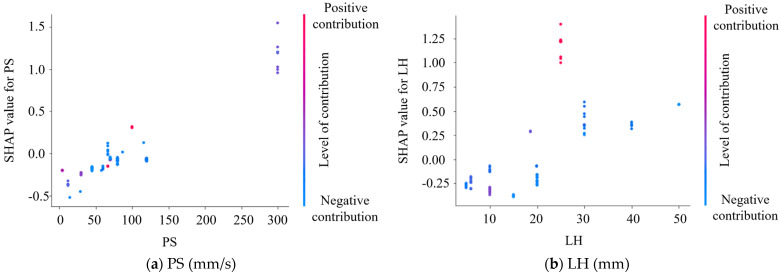

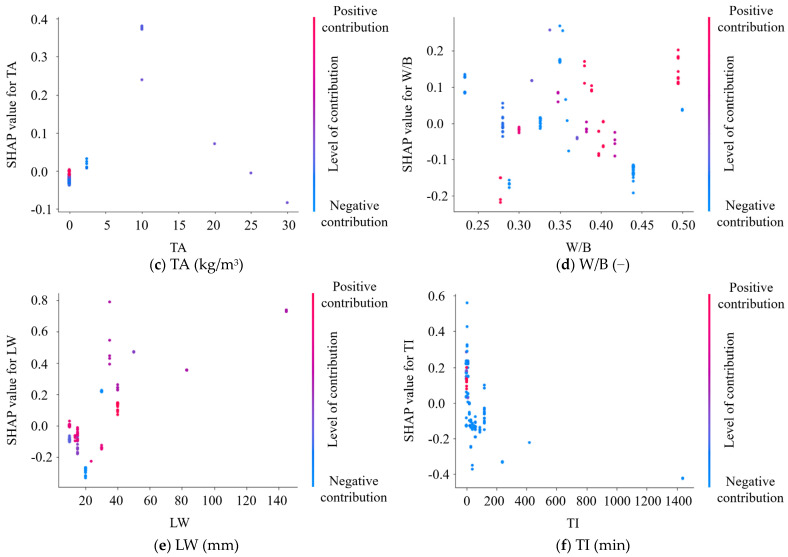
SHAP values of the most influential features based on the IBS prediction model.

Additionally, to provide a clearer insight into how these important features specifically impact the model’s predictive outcomes, this study conducted SHAP analysis, as depicted in Figure 13 for global interpretation. SHAP values are utilized to explain the contribution of input features to the final predictions of the model, aiding in understanding the predictive outcomes of the model. The global interpretation plot demonstrates the SHAP values of each important feature, providing consistent and fair explanations of feature contributions. Relative percentages of positive and negative contributions of each important feature are also observed in Figure 13 (where blue denotes negative contributions and red denotes positive contributions), consistent with Figure 12. Furthermore, to elucidate the reason why the model developed in this study is successful in predicting the IBS of 3D printing concrete, a further evaluation of the SHAP values of each important feature is conducted. Figure 14 illustrates the overall impact patterns of IBS. Regarding impact intensity, IBS increases with increases in PS and LW, decreases with increases in TI, exhibits a trend of initially increasing and then decreasing with increases in LH and TA, and shows a trend of decreasing–increasing–decreasing–increasing with increases in W/B. Regarding positive and negative contributions, both excessively high and low values of PS and LH yield negative contributions, with positive contributions observed only at intermediate values. This indicates that moderate PS and LH contribute to ensuring the more thorough deposition and curing of each layer, thereby enhancing interlayer bonding strength. For TA and TI, only lower values yield positive contributions, suggesting that adding some TA and setting TI lower enhances the deposition of concrete in each layer, strengthening interlayer bonding. As for W/B, both high and low values may result in positive or negative contributions, necessitating a consideration of actual material and printing parameters, such as larger W/B improving IBS when TI is higher, and smaller W/B enhancing IBS when TI is lower. Concerning LW, as long as the value is not excessively large, positive contributions may be observed, indicating that excessively large LW leads to uneven material distribution and irregular interlayer interface shapes, reducing bonding surface area and consequently lowering IBS. In practical applications, by finely adjusting these influencing factors, it is possible to optimize the IBS of concrete while considering printing efficiency, providing effective guidance for the preparation of high-quality 3D printing concrete structures.

## 5. Discussion and Future Prospects

Based on a database composed of typical data, this study establishes a prediction model for the IBS of 3D printing concrete using ML algorithms. In practical applications, such as experiments or construction processes, actual mix proportions and printing parameters can be inputted into the prediction model to obtain the IBS of 3D printing concrete, allowing for the prediction of printing outcomes in advance. If the predicted IBS is unsatisfactory, adjustments to the mix proportions or printing parameters can be made beforehand. This approach maximizes resource and cost savings while improving efficiency and accuracy. The content of this study is just a starting point, as the authors intend to conduct further experiments to validate the research findings and advance the realization of the aforementioned objectives.

This study focuses on establishing a prediction model for the IBS of 3D printing concrete, serving as a foundation for future design of mix proportions and printing parameters. The next step involves utilizing optimization algorithms (such as genetic algorithm—GA) to optimize the mix proportions and printing parameters of 3D printing concrete, aiming to achieve a specified IBS and more precise material formulations and printing processes. The main steps are roughly as follows:

(1) Prediction model establishment: Develop a prediction model for the IBS of 3D printing concrete (referred to as Model A) based on the methods employed in this study.
(2) Design of mix proportions and printing parameters: Employ GA for automatic inversion optimization. Building upon Model A, conduct single-objective optimization (e.g., setting the objective function as the specified IBS) or multi-objective optimization (e.g., setting the objective function as the specified IBS, lowest carbon emissions, or lowest cost). Through iteration, inversion, and optimization, the optimized mix proportions and printing parameters that meet the desired performance can be obtained.

This optimization design stage aims to provide stronger support for the practical application of 3D printing concrete, promoting sustainable development and technological innovation in the field. Furthermore, building upon the theoretical underpinnings of this study, future research endeavors will expand exploration into more sophisticated stacking models and other advanced ML methodologies. The objective is to delve into diverse model architectures and algorithmic combinations while staying abreast of the latest developments in ML techniques, ensuring a comprehensive assessment of their efficacy. By integrating automated 3D printing technology with intelligent ML methodologies, the aim is to offer technical backing and solutions for the architectural sector’s digital transformation.

To overcome some current limitations such as small data volumes and unreliable data sources, a novel approach is planned for the future. This approach involves coupling physics-based mechanistic models with data-driven ML models. This method aims to leverage the strengths of both approaches, allowing for professional constraints on ML models through mechanistic models, even with relatively limited data in the database. Additionally, there are plans to integrate data from the literature, experimental data, and data derived from mechanistic models to build a more comprehensive and robust database. By comprehensively utilizing these data and coupling models, the aim is to enhance the accuracy, generalization capability, and robustness of prediction models. This strategy holds promise for providing more reliable results when facing limited data and complexity issues.

## 6. Conclusions

This study developed a stacking learning model to predict the IBS of 3D printing concrete. A total of 146 data points are collected from various sources in the literature, and sixteen input parameters are utilized to establish the IBS prediction model. The main findings are as follows:

1. The base models, SVR, KNN, and GPR, demonstrate good predictive performance for the IBS of 3D printing concrete, with *R*^2^ ranging from 0.87 to 0.94. However, there remains potential for further enhancement.
2. The stacking model outperforms the base models in predicting the IBS of 3D printing concrete, achieving an *R*^2^ as high as 0.96. The performance remains consistent between the training and testing sets, highlighting its strong generalization capability.
3. A 10-fold cross-validation method is employed to compare the predictive effects of the base and stacking models. The stacking model exhibits the highest *R*^2^ compared to the base models, with relatively small fluctuations. Furthermore, statistical check indicators on the testing set revealed that the stacking model has the lowest *RMSE*. The high performance of the stacking model is attributed to the integration of the advantages of the three base models, offsetting their negative impacts and ultimately improving the overall predictive performance of the model.
4. Through importance analysis, it is determined that the top six factors significantly affecting the IBS of 3D printing concrete are PS, LH, TA, W/B, LW, and TI. In practical applications, adjustments to these influential factors can be made based on the prediction results, promoting the realization of high-quality 3D printing concrete structures.

## Figures and Tables

**Table 1:** The definition and range of hyperparameters of ML algorithms.

No.	Hyperparameters	Definition	Range	Search Interval
SVR	*c*	The penalty coefficient	100–1000	100
	*γ*	The gamma value of the Gaussian kernel	10–100	10
KNN	n_neighbors	The number of nearest neighbors	1–30	1
GPR	n_restarts_optimizer	The number of optimizer restarts	0–10	1

**Table 2:** Descriptive statistics of the model parameters used to predict IBS.

Descriptive Statistic	Input Parameters																Output Parameter
	OPC	SAC	SF	FA	NC	S	MAXSS	TA	ESA	SP/B	W/B	ICA	TI	PS	LH	LW	IBS
	(kg/m^3^)	(kg/m^3^)	(kg/m^3^)	(kg/m^3^)	(kg/m^3^)	(kg/m^3^)	(mm)	(kg/m^3^)	(kg/m^3^)	/	/	(kg/m^3^)	(min)	(mm/s)	(mm)	(mm)	(MPa)
M	797.90	67.91	89.29	95.70	30.54	1107.55	1.33	1.25	0.82	0.01	0.37	25.68	86.15	92.38	16.12	27.00	2.69
SD	307.63	79.98	104.65	285.52	94.73	475.25	1.45	4.31	1.34	0.02	0.08	48.18	262.11	72.62	10.73	24.73	2.22
Min	270.00	0.00	0.00	0.00	0.00	0.00	0.00	0.00	0.00	0.00	0.23	0.00	0.00	4.23	5.00	10.00	0.27
25%	500.00	0.00	0.00	0.00	0.00	850.00	0.60	0.00	0.00	0.00	0.30	0.00	2.00	48.25	10.00	15.00	0.94
50%	850.00	50.00	25.00	0.00	0.00	1180.00	0.80	0.00	0.00	0.00	0.37	0.00	20.00	70.00	10.00	20.00	1.90
75%	1000.00	120.00	200.00	0.00	11.20	1500.00	1.20	0.00	1.60	0.00	0.44	25.00	60.00	120.00	20.00	35.00	3.67
Max	1400.00	405.00	250.00	1000.00	400.00	1750.00	5.00	30.00	3.30	0.16	0.50	160.00	1440.00	300.00	50.00	145.00	11.00
C	146	146	146	146	146	146	146	146	146	146	146	146	146	146	146	146	146

**Table 3:** Prediction results of the base and stacking models on the testing set.

Type	Model	*R* ^2^	*RMSE*
		(−)	(MPa)
Base models	SVR	0.94	0.61
	KNN	0.92	0.61
	GPR	0.87	0.87
Stacking model	SVR + KNN + GPR	0.96	0.39

## Data Availability

Data are contained within the article.

## Supplementary Materials

The following supporting information can be downloaded at: https://www.mdpi.com/article/10.3390/ma17051033/s1, Table S1: Database of the IBS prediction model.

## Abbreviations

IBS	interlayer bonding strength	NC	nano clay
ML	machine learning	S	sand
SVR	support vector regression	MAXSS	maximum sand particle size
KNN	k-nearest neighbor	TA	thixotropic agent
GPR	Gaussian process regression	ESA	early strength agent
ANN	artificial neural network	SP/B	superplasticizer/binder
GEP	gene expression programming	W/B	water/binder
SVM	support vector machine	ICA	interlayer bonding agent
LR	linear regression	TI	time interval
DT	decision tree	PS	printing speed
RF	random forest	LH	printing layer height
Ridge	ridge regression	LW	printing layer width
Lasso	least absolute shrinkage and selection operator	M	mean
OLS	ordinary least squares	SD	standard deviation
XGBoost	extreme gradient boosting	Min	minimum
stacking	SVR + KNN + GPR	Max	maximum
GA	genetic algorithm	C	count
OPC	ordinary Portland cement	*R*	correlation coefficient
SAC	sulphate aluminate cement	*R* ^2^	coefficient of determination
SF	silica fume	*RMSE*	root mean square error
FA	fly ash	SHAP	Shapley additive explanations
