# Supplementary material for: Advancing Construction 3D Printing with Predictive Interlayer Bonding Strength: A Stacking Model Paradigm
Source: Materials (Basel). 2024 Feb 23;17(5):1033. doi: 10.3390/ma17051033 (PMC10935051; doi:10.3390/ma17051033)
Supplement: Supplementary file 1 [file materials-17-01033-s001.zip › materials-2873430-supplementary.pdf]

Table S1. Database of the IBS prediction model.

OPC	SAC	SF	FA	NC	S	MAXSS	TA	ESA	SP/B	W/B	ICA	TI	PS	LH	LW	IBS	Reference
900	100	0	0	0	0	0	10	1.6	0.0006	0.35	0	0	300	25	30	10	[41]
900	100	0	0	0	0	0	10	1.6	0.0006	0.35	0	0	300	25	30	8	
900	100	0	0	0	0	0	10	1.6	0.0006	0.35	0	0	300	25	30	7.8	
900	100	0	0	0	0	0	10	1.6	0.0006	0.35	0	0	300	25	30	7.7	
900	100	0	0	0	0	0	10	1.6	0.0006	0.35	0	0	300	25	30	7.6	
900	100	0	0	0	0	0	10	1.6	0.0006	0.35	0	0	300	25	30	7.5	
900	100	0	0	0	1200	1.2	0	1.6	0	0.35	0	5	300	25	35	11	[42]
895	100	0	0	0	1200	1.2	5	1.6	0.000201	0.351759	0	5	300	25	35	9	
890	100	0	0	0	1200	1.2	10	1.6	0.000505	0.353535	0	5	300	25	35	8.5	
885	100	0	0	0	1200	1.2	15	1.6	0.000812	0.35533	0	5	300	25	35	7.8	
880	100	0	0	0	1200	1.2	20	1.6	0.001429	0.357143	0	5	300	25	35	7	
875	100	0	0	0	1200	1.2	25	1.6	0.001949	0.358974	0	5	300	25	35	6.5	
870	100	0	0	0	1200	1.2	30	1.6	0.002371	0.360825	0	5	300	25	35	5.5	
500	0	0	0	0	500	0.6	0	3.3	0	0.44	0	0	120	10	20	1.07	[43]
470	30	0	0	0	500	0.6	0	3.3	0	0.44	0	0	120	10	20	0.94	
450	50	0	0	0	500	0.6	0	3.3	0	0.44	0	0	120	10	20	0.72	
420	80	0	0	0	500	0.6	0	3.3	0	0.44	0	0	120	10	20	0.56	
390	110	0	0	0	500	0.6	0	3.3	0	0.44	0	0	120	10	20	0.37	
500	0	0	0	0	500	0.6	0	3.3	0	0.44	0	10	120	10	20	1.07	
470	30	0	0	0	500	0.6	0	3.3	0	0.44	0	10	120	10	20	0.94	
450	50	0	0	0	500	0.6	0	3.3	0	0.44	0	10	120	10	20	0.72	

420	80	0	0	0	500	0.6	0	3.3	0	0.44	0	10	120	10	20	0.56	
390	110	0	0	0	500	0.6	0	3.3	0	0.44	0	10	120	10	20	0.37	
500	0	0	0	0	500	0.6	0	3.3	0	0.44	0	20	120	10	20	1.07	
470	30	0	0	0	500	0.6	0	3.3	0	0.44	0	20	120	10	20	0.94	
450	50	0	0	0	500	0.6	0	3.3	0	0.44	0	20	120	10	20	0.72	
420	80	0	0	0	500	0.6	0	3.3	0	0.44	0	20	120	10	20	0.56	
390	110	0	0	0	500	0.6	0	3.3	0	0.44	0	20	120	10	20	0.37	
500	0	0	0	0	500	0.6	0	3.3	0	0.44	0	30	120	10	20	1.07	
470	30	0	0	0	500	0.6	0	3.3	0	0.44	0	30	120	10	20	0.94	
450	50	0	0	0	500	0.6	0	3.3	0	0.44	0	30	120	10	20	0.72	
420	80	0	0	0	500	0.6	0	3.3	0	0.44	0	30	120	10	20	0.56	
390	110	0	0	0	500	0.6	0	3.3	0	0.44	0	30	120	10	20	0.37	
500	0	0	0	0	500	0.6	0	3.3	0	0.44	0	40	120	10	20	1.07	
470	30	0	0	0	500	0.6	0	3.3	0	0.44	0	40	120	10	20	0.94	
450	50	0	0	0	500	0.6	0	3.3	0	0.44	0	40	120	10	20	0.72	
420	80	0	0	0	500	0.6	0	3.3	0	0.44	0	40	120	10	20	0.56	
390	110	0	0	0	500	0.6	0	3.3	0	0.44	0	40	120	10	20	0.37	
500	0	0	0	0	500	0.6	0	3.3	0	0.44	0	50	120	10	20	1.07	
470	30	0	0	0	500	0.6	0	3.3	0	0.44	0	50	120	10	20	0.94	
450	50	0	0	0	500	0.6	0	3.3	0	0.44	0	50	120	10	20	0.72	
420	80	0	0	0	500	0.6	0	3.3	0	0.44	0	50	120	10	20	0.56	
390	110	0	0	0	500	0.6	0	3.3	0	0.44	0	50	120	10	20	0.37	
1000	0	250	0	0	1500	1	0	0	0	0.495	25	0.25	70	10	10	3.68	[7]
1000	0	250	0	0	1500	1	0	0	0	0.495	25	60	70	10	10	3.51	

1000	0	250	0	0	1500	1	0	0	0	0.495	25	240	70	10	10	3.48	
1000	0	250	0	0	1500	1	0	0	0	0.495	25	420	70	10	10	3.34	
1000	0	250	0	0	1500	1	0	0	0	0.495	25	1440	70	10	10	3.1	
1000	0	250	0	0	1500	1	0	0	0	0.495	25	0.25	70	10	10	3.92	
1000	0	250	0	0	1500	1	0	0	0	0.495	25	0.25	70	10	10	3.98	
1000	0	250	0	0	1500	1	0	0	0	0.495	25	1440	70	10	10	2.65	
1000	0	250	0	0	1500	1	0	0	0	0.495	25	1440	70	10	10	2.35	
1000	0	250	0	0	1500	1	0	0	0	0.495	25	240	70	10	10	1.72	
1000	0	250	0	0	1500	1	0	0	0	0.495	25	1440	70	10	10	2.28	
1000	0	250	0	0	1500	1	0	0	0	0.495	25	0.25	70	10	10	3.55	
1000	0	250	0	0	1500	1	0	0	0	0.495	25	240	70	10	10	3.45	
1000	0	250	0	0	1500	1	0	0	0	0.495	25	1440	70	10	10	2.8	
500	160	25	0	0	1180	2	0	0	0.007299	0.233577	25	24	66.7	19.9	83	2.1	[5]
500	160	25	0	0	1180	2	0	0	0.007299	0.233577	25	24	66.7	19.9	83	3.65	
500	160	25	0	0	1180	2	0	0	0.007299	0.233577	25	24	66.7	19.9	83	4.05	
500	160	25	0	0	1180	2	0	0	0.007299	0.233577	25	24	66.7	19.9	83	5.01	
500	160	25	0	0	1180	2	0	0	0.007299	0.233577	25	10	100	18.6	145	5.05	
500	160	25	0	0	1180	2	0	0	0.007299	0.233577	25	10	100	18.6	145	5.55	
500	160	25	0	0	1180	2	0	0	0.007299	0.233577	25	10	100	18.6	145	6.1	
500	160	25	0	0	1180	2	0	0	0.007299	0.233577	25	10	100	18.6	145	7.1	
568	0	81	162	0	1175	0.33	0	0	0.006	0.31566	0	0	70	10	40	3.95	[8]
568	0	81	162	0	1175	0.33	0	0	0.006	0.31566	0	0	70	10	40	3.55	
568	0	81	162	0	1175	0.33	0	0	0.006	0.337707	2.433	0	70	10	40	4.75	
568	0	81	162	0	1175	0.33	0	0	0.006	0.337707	2.433	0	70	10	40	4.4	

568	0	81	162	0	1175	0.33	0	0	0.006	0.371147	2.433	0	70	10	40	3.8	
568	0	81	162	0	1175	0.33	0	0	0.006	0.371147	2.433	0	70	10	40	3.3	
270	405	0	0	0	1012.5	4.75	0	0	0	0.5	0	12	4.23	50	50	4.73	
270	405	0	0	0	1012.5	4.75	0	0	0	0.5	0	12	4.23	50	50	4.62	[44]
270	405	0	0	0	1012.5	4.75	0	0	0	0.5	0	12	4.23	50	50	4.36	
1000	0	0	0	250	1500	0.8	0	0	0	0.288	0	0	12	15	30	0.27	
1000	0	0	0	250	1500	0.8	0	0	0	0.288	0	0	12	15	30	0.3	
1000	0	0	0	250	1500	0.8	0	0	0	0.288	3	0	12	15	30	0.34	[45]
1000	0	0	0	250	1500	0.8	0	0	0	0.288	6	0	12	15	30	0.35	
1000	0	0	0	250	1500	0.8	0	0	0.0024	0.288	0	0	12	15	30	0.425	
850	150	250	0	12.4	1500	0.8	0	0	0.003565	0.388942	80	60	80	6	15	4.3	
850	150	250	0	12.4	1500	0.8	0	0	0.003565	0.380228	160	60	80	6	15	4	
850	150	250	0	12.4	1500	0.8	0	0	0.003565	0.4032	100	60	80	6	15	2.25	
850	150	250	0	12.4	1500	0.8	0	0	0.003565	0.397655	150	60	80	6	15	1.5	
850	150	250	0	12.4	1500	0.8	0	0	0.003565	0.27725	0	60	80	6	15	1.3	
850	150	250	0	12.4	1500	0.8	0	0	0.003565	0.388942	80	120	80	6	15	4.2	
850	150	250	0	12.4	1500	0.8	0	0	0.003565	0.380228	160	120	80	6	15	4	[40]
850	150	250	0	12.4	1500	0.8	0	0	0.003565	0.4032	100	120	80	6	15	1.75	
850	150	250	0	12.4	1500	0.8	0	0	0.003565	0.397655	150	120	80	6	15	1.45	
850	150	250	0	12.4	1500	0.8	0	0	0.003565	0.27725	0	120	80	6	15	1.1	
850	150	250	0	12.4	1500	0.8	0	0	0.003565	0.388942	80	60	80	6	15	1.5	
850	150	250	0	12.4	1500	0.8	0	0	0.003565	0.380228	160	60	80	6	15	1.75	
850	150	250	0	12.4	1500	0.8	0	0	0.003565	0.4032	100	60	80	6	15	1.6	
850	150	250	0	12.4	1500	0.8	0	0	0.003565	0.397655	150	60	80	6	15	2.05	

850	150	250	0	12.4	1500	0.8	0	0	0.003565	0.27725	0	60	80	6	15	0.5	
850	150	250	0	12.4	1500	0.8	0	0	0.003565	0.388942	80	120	80	6	15	1.8	
850	150	250	0	12.4	1500	0.8	0	0	0.003565	0.380228	160	120	80	6	15	1.75	
850	150	250	0	12.4	1500	0.8	0	0	0.003565	0.4032	100	120	80	6	15	2.2	
850	150	250	0	12.4	1500	0.8	0	0	0.003565	0.397655	150	120	80	6	15	2.15	
850	150	250	0	12.4	1500	0.8	0	0	0.003565	0.27725	0	120	80	6	15	0.7	
1400	0	0	0	9.8	1750	5	0	0	0.002979	0.326287	0	0	30	20	40	1.86	
1400	0	0	0	11.2	1750	5	0	0	0.002976	0.325964	0	0	30	20	40	1.88	
1400	0	0	0	11.2	1750	5	0	0	0.002976	0.325964	0	0	30	20	40	1.96	
1400	0	0	0	9.8	1750	5	0	0	0.002979	0.326287	0	10	30	20	40	1.72	
1400	0	0	0	11.2	1750	5	0	0	0.002976	0.325964	0	10	30	20	40	1.6	
1400	0	0	0	11.2	1750	5	0	0	0.002976	0.325964	0	10	30	20	40	1.9	
1400	0	0	0	9.8	1750	5	0	0	0.002979	0.326287	0	20	30	20	40	1.52	
1400	0	0	0	11.2	1750	5	0	0	0.002976	0.325964	0	20	30	20	40	1.25	[46]
1400	0	0	0	11.2	1750	5	0	0	0.002976	0.325964	0	20	30	20	40	1.65	
1400	0	0	0	9.8	1750	5	0	0	0.002979	0.326287	0	30	30	20	40	0.88	
1400	0	0	0	11.2	1750	5	0	0	0.002976	0.325964	0	30	30	20	40	0.77	
1400	0	0	0	11.2	1750	5	0	0	0.002976	0.325964	0	30	30	20	40	0.94	
1400	0	0	0	9.8	1750	5	0	0	0.002979	0.326287	0	40	30	20	40	0.64	
1400	0	0	0	11.2	1750	5	0	0	0.002976	0.325964	0	40	30	20	40	0.55	
1400	0	0	0	11.2	1750	5	0	0	0.002976	0.325964	0	40	30	20	40	0.67	
400	0	200	0	400	1500	2	2.4	0	0.02	0.3	0	0	60	40	13.5	3.05	
400	0	200	0	400	1500	2	2.4	0	0.02	0.3	0	0.2	60	40	13.5	3.5	[47]
400	0	200	0	400	1500	2	2.4	0	0.02	0.3	0	1	60	40	13.5	2.95	

400	0	200	0	400	1500	2	2.4	0	0.02	0.3	0	10	60	40	13.5	2.63	
400	0	200	0	400	1500	2	2.4	0	0.02	0.3	0	1	60	40	13.5	2.9	
400	0	200	0	400	1500	2	2.4	0	0.02	0.3	0	1	60	40	18.5	2.85	
400	0	200	0	400	1500	2	2.4	0	0.02	0.3	0	1	60	40	23.5	2.6	
910	90	150	0	0	850	0.8	0	0	0.002435	0.347826	80	60	45	5	10	1.6	[48]
910	90	150	0	0	850	0.8	0	0	0.002435	0.347826	160	60	45	5	10	1.9	
880	120	150	0	0	850	0.8	0	0	0.002435	0.347826	100	60	45	5	10	1.9	
880	120	150	0	0	850	0.8	0	0	0.002435	0.347826	150	60	45	5	10	1.8	
880	120	150	0	0	850	0.8	0	0	0.002435	0.382609	0	90	45	5	10	1.5	
880	120	150	0	0	850	0.8	0	0	0.002435	0.382609	80	90	45	5	10	1.8	
850	150	150	0	0	850	0.8	0	0	0.002435	0.382609	160	90	45	5	10	1.75	
850	150	150	0	0	850	0.8	0	0	0.002435	0.382609	100	90	45	5	10	1.55	
880	120	150	0	0	850	0.8	0	0	0.002435	0.417391	150	120	45	5	10	1.7	
880	120	150	0	0	850	0.8	0	0	0.002435	0.417391	0	120	45	5	10	1.8	
850	150	150	0	0	850	0.8	0	0	0.002435	0.417391	80	120	45	5	10	1.75	
850	150	150	0	0	850	0.8	0	0	0.002435	0.417391	160	120	45	5	10	0.9	
1000	0	50	1000	0	1025	0.25	0	0	0.078049	0.28	0	2	66.7	30	15	3.09	[49]
1000	0	50	1000	0	1025	0.25	0	0	0.097561	0.28	0	2	66.7	30	15	3.74	
1000	0	50	1000	0	1025	0.25	0	0	0.117073	0.28	0	2	66.7	30	15	4.16	
1000	0	50	1000	0	1025	0.25	0	0	0.136585	0.28	0	2	66.7	30	15	4.73	
1000	0	50	1000	0	1025	0.25	0	0	0.156098	0.28	0	2	66.7	30	15	5.39	
1000	0	50	1000	0	1025	0.25	0	0	0.058537	0.28	0	2	14.5	30	15	1.9	
1000	0	50	1000	0	1025	0.25	0	0	0.058537	0.28	0	2	29	30	15	2.25	
1000	0	50	1000	0	1025	0.25	0	0	0.058537	0.28	0	2	58	30	15	2.56	

1000	0	50	1000	0	1025	0.25	0	0	0.058537	0.28	0	2	87	30	15	2.84	
1000	0	50	1000	0	1025	0.25	0	0	0.058537	0.28	0	2	116	30	15	3	
1000	0	50	1000	0	1025	0.25	0	0	0.058537	0.28	0	2	66.7	30	15	2.6	
1000	0	50	1000	0	1025	0.25	0	0	0.058537	0.28	0	2	66.7	30	15	3.08	
1000	0	50	1000	0	1025	0.25	0	0	0.058537	0.28	0	2	66.7	30	15	3.51	

References

5 Kloft, H.; Krauss, H.-W.; Hack, N.; Herrmann, E.; Neudecker, S.; Varady, P.A.; Lowke, D. Influence of Process Parameters on the Interlayer Bond Strength of Concrete Elements Additive Manufactured by Shotcrete 3D Printing (SC3DP). *Cem Concr Res* **2020**, *134*, 106078. https://doi.org/10.1016/j.cemconres.2020.106078.

7 Wolfs, R.J.M.; Bos, F.P.; Salet, T.A.M. Hardened Properties of 3D Printed Concrete: The Influence of Process Parameters on Interlayer Adhesion. *Cem Concr Res* **2019**, *119*, 132–8 140. https://doi.org/10.1016/j.cemconres.2019.02.017.

Moelich, G.M.; Kruger, J.; Combrinck, R. Modelling the Interlayer Bond Strength of 3D Printed Concrete with Surface Moisture. *Cem Concr Res* **2021**, *150*, 106559. https://doi.org/10.1016/j.cemconres.2021.106559.

40 Wang, L.; Tian, Z.; Ma, G.; Zhang, M. Interlayer Bonding Improvement of 3D Printed Concrete with Polymer Modified Mortar: Experiments and Molecular Dynamics Studies. *Cem Concr Compos* **2020**, *110*, 103571. https://doi.org/10.1016/j.cemconcomp.2020.103571.

41 Xu, Y.; Yuan, Q.; Li, Z.; Shi, C.; Wu, Q.; Huang, Y. Correlation of Interlayer Properties and Rheological Behaviors of 3DPC with Various Printing Time Intervals. *Addit Manuf* **2021**, *47*, 102327. https://doi.org/10.1016/j.addma.2021.102327.

42 Yao, H.; Xie, Z.; Li, Z.; Huang, C.; Yuan, Q.; Zheng, X. The Relationship between the Rheological Behavior and Interlayer Bonding Properties of 3D Printing Cementitious Materials with the Addition of Attapulgite. *Constr Build Mater* **2022**, *316*, 125809. https://doi.org/10.1016/j.conbuildmat.2021.125809.

43 Liu, C.; Xiong, Y.; Chen, Y.; Jia, L.; Ma, L.; Deng, Z.; Wang, Z.; Chen, C.; Banthia, N.; Zhang, Y. Effect of Sulphoaluminate Cement on Fresh and Hardened Properties of 3D Printing Foamed Concrete. *Compos B Eng* **2022**, *232*, 109619. https://doi.org/10.1016/j.compositesb.2022.109619.

44 Zareiyan, B.; Khoshnevis, B. Effects of Interlocking on Interlayer Adhesion and Strength of Structures in 3D Printing of Concrete. *Autom Constr* **2017**, *83*, 212–221. https://doi.org/10.1016/j.autcon.2017.08.019.

45 Marchment, T.; Sanjayan, J.; Xia, M. Method of Enhancing Interlayer Bond Strength in Construction Scale 3D Printing with Mortar by Effective Bond Area Amplification. *Mater Des* **2019**, *169*, 107684. https://doi.org/10.1016/j.matdes.2019.107684.

46 Pan, T.; Jiang, Y.; He, H.; Wang, Y.; Yin, K. Effect of Structural Build-up on Interlayer Bond Strength of 3D Printed Cement Mortars. *Materials* **2021**, *14*, 236.

47 Chen, Y.; Jansen, K.; Zhang, H.; Romero Rodriguez, C.; Gan, Y.; Çopuroğlu, O.; Schlangen, E. Effect of Printing Parameters on Interlayer Bond Strength of 3D Printed Limestone-Calcined Clay-Based Cementitious Materials: An Experimental and Numerical Study. *Constr Build Mater* **2020**, *262*, 120094. https://doi.org/10.1016/j.conbuildmat.2020.120094.

48 Ma, G.; Salman, N.M.; Wang, L.; Wang, F. A Novel Additive Mortar Leveraging Internal Curing for Enhancing Interlayer Bonding of Cementitious Composite for 3D Printing. *Constr Build Mater* **2020**, *244*, 118305. https://doi.org/10.1016/j.conbuildmat.2020.118305.

49 Weng, Y.; Li, M.; Zhang, D.; Tan, M.J.; Qian, S. Investigation of Interlayer Adhesion of 3D Printable Cementitious Material from the Aspect of Printing Process. *Cem Concr Res* **2021**, *143*, 106386. https://doi.org/10.1016/j.cemconres.2021.106386.
